# Novel Hybrid Composites Based on Polymers of Diphenyl-Amine-2-Carboxylic Acid and Highly Porous Activated IR-Pyrolyzed Polyacrylonitrile

**DOI:** 10.3390/polym15020441

**Published:** 2023-01-13

**Authors:** Sveta Zhiraslanovna Ozkan, Valeriy Alekseevich Petrov, Mikhail Nikolaevich Efimov, Andrey Aleksandrovich Vasilev, Dmitriy Gennad’evich Muratov, Alexey Aleksandrovich Sadovnikov, Galina Nikolaevna Bondarenko, Galina Petrovna Karpacheva

**Affiliations:** A.V. Topchiev Institute of Petrochemical Synthesis, Russian Academy of Sciences, 29 Leninsky Prospect, Moscow 119991, Russia

**Keywords:** polydiphenylamine-2-carboxylic acid, in situ oxidative polymerization, polymer–carbon composites, activated IR-pyrolyzed polyacrylonitrile, IR heating

## Abstract

Hybrid composites based on electroactive polymers of diphenylamine-2-carboxylic acid (PDPAC) and highly porous carbon with a hierarchical pore structure were prepared for the first time. Activated IR-pyrolyzed polyacrylonitrile (IR-PAN-a), characterized by a highly developed surface, was chosen as a highly porous N-doped carbon component of the hybrid materials. IR-PAN-a was prepared using pyrolysis of polyacrylonitrile (PAN) in the presence of potassium hydroxide under IR radiation. Composite materials were obtained using oxidative polymerization of diphenylamine-2-carboxylic acid (DPAC) in the presence of IR-PAN-a both in an acidic and an alkaline medium. The composite materials were IR-heated to reduce the oxygen content and enhance their physical and chemical properties. The chemical structure, morphology, and electrical and thermal properties of the developed IR-PAN-a/PDPAC composites were investigated. The IR-PAN-a/PDPAC composites are thermally stable and electrically conductive. During the synthesis of the composites in an acidic medium, doping of the polymer component occurs, which makes the main contribution to the composite conductivity (1.3 × 10^–5^ S/cm). A sharp drop in the electrical conductivity of the IR-PAN-a/PDPAC_ac-IR_ composites to 3.4 × 10^–10^ S/cm is associated with the removal of the dopant during IR heating. The IR-PAN-a/PDPAC_alk_ composites prepared before and after IR heating show a gradual increase in electrical conductivity by five orders of magnitude to 1.6 × 10^–5^ S/cm at 25–10^6^ Hz. IR heating of the obtained materials leads to a significant increase in their thermal properties. The IR-heated composites lose half of their initial weight in an inert atmosphere at temperatures above 1000 °C, whereas for IR-PAN-a/PDPAC, the temperature range is 840–849 °C.

## 1. Introduction

One of the priority areas for the technological development strategy is the transition to resource-saving energy, as well as the creation of new ways to store energy. The rapid growth of electricity generation, its escalating use in transport, and the popularity of wearable electronic devices cause interest in the development of more efficient energy storage systems [1,2,3,4,5,6,7].

Today, the best results are obtained in the domain of hybrid supercapacitors, where energy is stored using the mechanism of electrostatic charging of the electrical double layer (double-layer capacitance) and fast and reversible Faraday reactions (pseudocapacitance) at the electrode/electrolyte interface. This can be achieved using the choice and optimal combination of electrode material components, such as carbon nanomaterials and conductive polymers [8,9,10,11,12,13]. The rapid transport of electrolyte ions in the porous electrode material ensures the achievement of high charge and discharge current densities. Composite materials based on conjugated polymers and carbon nanomaterials are promising for the creation of electrochemical current sources, supercapacitors, rechargeable batteries, low-temperature fuel cells, and solar panels, for the remediation of water resources, etc. [14,15,16,17,18,19,20,21,22,23].

Nowadays, many methods for the production of nanocomposites based on conjugated polymers and carbon materials as electroactive coatings for hybrid electrodes have been developed [24,25]. Oxidative polymerization of the monomer on the surface of carbon materials, which allows for obtaining a homogeneous polymer coating, seems to be the most promising [26,27]. Polyaniline, which is prepared using the oxidative polymerization of aniline in aqueous solutions of acids, is the most common of these in use. Widening the scope of electroactive polymers is hampered by the limited solubility of new monomers in aqueous solutions of acids.

Carbon nanotubes and grapheme-like materials can be used as carbon materials for hybrid electrode components [28,29,30,31]. Earlier, polymer–carbon hybrid nanomaterials based on poly-3-amine-7-methylamine-2-methylphenazine (PAMMP), polydiphenylamine-2-carboxylic acid (PDPAC), and single-walled carbon nanotubes (SWCNT) were obtained [32,33]. The synthesis of nanocomposites was carried out using oxidative polymerization of monomers in the presence of SWCNT. The resulting SWCNT/PAMMP and SWCNT/PDPAC nanocomposites are thermally stable and electrically conductive. Electrochemical properties of electrode coatings made of SWCNT/PDPAC nanocomposites in an organic lithium aprotic electrolyte in propylene carbonate were investigated [34]. The specific capacitance of electroactive coatings on anodized graphite foil (AGF) in an acidic medium reaches 438, 350, and 259 F∙g^−1^, whereas in an alkaline medium capacitance is 278, 191, and 129 F∙g^−1^ at charge–discharge currents of 0.5, 1.5, and 3.0 mА·cm^–2^, respectively. The high electrochemical capacitance of electrode materials in an organic electrolyte makes them promising as a cathode material for SC.

The introduction of sulfur, phosphorus, and nitrogen heteroatoms into the structure of graphene-like materials plays an important role. It is assumed that the N-doping of graphene oxide leads to the distortion of graphene nanosheets and an increase in the surface area [35,36]. The crumpled structure of nanosheets leads to an increase in the interplanar distance and, consequently, in the free space for electrolyte placement, providing high accessibility of electrolyte ions to the active surface.

The use of activated IR-pyrolyzed polyacrylonitrile (IR-PAN-a), prepared by the authors for the first time, offers exciting possibilities for setting a target for the electrochemical properties of resulting composites by changing the structural characteristics of the carbon material. The carbon structure of IR-PAN-a contains nitrogen atoms that provide additional capacity. The electrochemical behavior of composite coatings based on polyaniline (PANI) and IR-PAN-a on a loosened AGF surface has been studied. The IR-PAN-a/PANI coatings on AGF were obtained using electrochemical polymerization in a 1 M H_2_SO_4_ solution electrolyte. The IR-PAN-a/PANI composite coatings on AGF in the H_2_SO_4_ solution electrolyte are characterized by high values of specific electrochemical capacity (C_s_ = 2.8 F∙cm^–2^) at 100% Coulomb efficiency and by high stability during long-term cycling processes [37].

In the present paper, hybrid composites based on PDPAC and IR-PAN-a as highly porous N-doped carbon components were prepared for the first time. Hybrid IR-PAN-a/PDPAC materials were synthesized using in situ oxidative polymerization of diphenylamine-2-carboxylic acid (DPAC) in the presence of IR-PAN-a in an acidic and an alkaline medium. The resulting composite materials were IR-heated to reduce the oxygen content and enhance their physical and chemical properties. The structure, morphology, and thermal and electrical properties of the obtained composites were investigated.

The choice of polymer is due to the fact that PDPAC is a novel conjugated polyacid synthesized by the authors for the first time. The chemical structure of PDPAC has a strong dependence on the pH of the reaction medium. The presence of the NH and COOH functional groups can be expected to determine the electronic interaction between the carbon nanomaterial and polymer not only through the main polymer chain but also through side substitutes. In addition, the presence of active carboxyl groups in PDPAC improves its solubility relative to the solubility of PANI.

## 2. Experimental Methods

### 2.1. Materials

Diphenylamine-2-carboxylic acid (DPAC) (C_13_H_11_O_2_N) (analytical grade), sulfuric acid (reagent grade), aqueous ammonia (reagent grade), and chloroform (reagent grade) were used without any additional purification. Ammonium persulfate (analytical grade) was purified using recrystallization from distilled water.

### 2.2. Synthesis of Activated IR-Pyrolyzed Polyacrylonitrile (IR-PAN-a)

Polyacrylonitrile (PAN) was IR heated at 200 °C in the air for 20 min. The suspension of IR-heated PAN in the KOH aqueous solution was exposed for 24 h and dried at 80 °C in a vacuum. The prepared powder was IR heated at 800 °C for 2 min in a nitrogen atmosphere. The heat treatment was conducted in a laboratory quartz tube IR furnace. The prepared IR-PAN-a was characterized by a microporous structure. The specific surface area of IR-PAN-a is 2438 m^2^∙g^−1^ [38].

### 2.3. Synthesis of PDPAC and IR-PAN-a/PDPAC

Polydiphenylamine-2-carboxylic acid was prepared using oxidative polymerization of DPAC in the homogeneous acidic medium (PDPAC_ac_) and the heterophase system in an alkaline medium (PDPAC_alk_). According to the GPC, in NH_4_OH (pH 11.4), the molecular weight reached *М_w_* = 2.6 × 10^4^ [39], whereas in 5 М H_2_SO_4_ (pH 0.3), the molecular weight is *М_w_* = 1.1 × 10^4^ [40].

IR-PAN-a/PDPAC composites were prepared using two methods, as well as PDPAC, as follows:

For the composite synthesis in an acidic medium (IR-PAN-a/PDPAC_ac_), first, IR-PAN-a was added to the DPAC solution (0.1 mol/L, 0.64 g) in 5 М H_2_SO_4_ and stirred in an ultrasonic bath (UZV-2414, Vologda, Russia) at room temperature for 0.5 h. The content of IR-PAN-a was С_IR-PAN-a_ = 3 wt % (0.0192 g) and 10 wt % (0.064 g) relative to the monomer weight. Then, the ammonium persulfate solution (0.2 mol/L, 1.368 g) in the same solvent (V_total_ = 30 mL) was added dropwise under intensive stirring to the IR-PAN-a/DPAC suspension, which was pre-cooled to 0 °C. The synthesis continued for 3 h with intense stirring at 0 °C. The reaction mixture was precipitated in 200 mL of distilled water. The resulting product was filtered off and washed repeatedly with a 1% solution of H_2_SO_4_. The yield of the IR-PAN-a/PDPAC_ac_ composite was 0.54 g (81.4%) at C_IR-PAN-a_ = 3 wt %.

For the composite synthesis in the heterophase system in an alkaline medium (IR-PAN-a/PDPAC_alk_), first, IR-PAN-a was added to the DPAC solution (0.1 mol/L, 0.64 g) in a mixture of chloroform (15 mL) and NH_4_OH (0.5 mol/L, 2.3 mL). The content of IR-PAN-a was С_IR-PAN-a_ = 3 wt % (0.0192 g) and 10 wt % (0.064 g) relative to the monomer weight. The IR-PAN-a/DPAC suspension stirring was carried out in an ultrasonic bath at room temperature for 0.5 h. Then, an aqueous solution (15 mL) of ammonium persulfate (0.2 mol/L, 1.368 g) was added in one go, without gradual dosing of reagents to the IR-PAN-a/DPAC suspension, which was pre-cooled to 0 °C (V_total_ = 30 mL). The synthesis was carried out for 3 h at 0 °C under intensive stirring. The mixture was precipitated in a tenfold excess of a 2% solution of H_2_SO_4_. The resulting product was filtered off and washed repeatedly with distilled water. The yield of IR-PAN-a/PDPAC_alk_ composite was 0.50 g (75.4%) with C_IR-PAN-a_ = 3 wt %.

### 2.4. IR Heating of IR-PAN-a/PDPAC Composites

The prepared IR-PAN-a/PDPAC composites were IR-heated with an automated IR heating unit [38] in a nitrogen atmosphere at 300 and 350 °C for 10 min. The IR heating temperature was determined using TGA data to prevent polymer chain degradation. The heating rate was 50 °C min^−1^. The composites were marked as IR-PAN-a/PDPAC_ac-IR_ and IR-PAN-a/PDPAC_alk-IR_.

### 2.5. Materials Characterization

Attenuated total reflection (ATR) FTIR spectra were recorded using a HYPERION-2000 IR microscope (Bruker, Karlsruhe, Germany) coupled with the Bruker IFS 66v FTIR spectrometer (Karlsruhe, Germany) in the range of 600–4000 cm^−1^ (ZnSe crystal, resolution of 2 cm^−1^).

The Raman spectra were recorded using a Senterra II Raman spectrometer (Bruker, Karlsruhe, Germany). A laser with a wavelength of 532 nm and a power of 0.25 mW was used. The spectral resolution was 4 cm^−1^.

The solid-state CP/MAS ^13^C NMR spectra were registered with a Bruker AVANCE II 400 WB spectrometer in a magnetic field of 9.4 T using a two-channel magic angle spinning (MAS) Bruker H/X probe with an outer diameter of 4 mm and rotation speed of 10 kHz. The chemical shifts of ^13^С nuclei were identified against the standard tetramethylsilane Si(CH_3_)_4_. The spectra were recorded using a cross-polarization pulse sequence (CP/MAS) with polarization transfer from ^1^H nuclei to ^13^С nuclei and decoupling over ^1^H nuclei. The duration of a ^1^H impulse was 3 µs, the contact time was 2 ms, and the time between scans was 3 s. The TopSpin 2.1 program (Bruker) was used to process the recorded spectra.

The high-resolution XPS spectra were recorded using a «PREVAC EA15» electron spectrometer (Rogów, Poland) equipped with an aluminum anode X-ray tube (AlKα radiation, hν = 1486.6 eV). The source power was 150 W. The pressure in the analytical chamber was 5·10^–9^ mbar. The binding energy scale was pre-calibrated using the positions of Ag3d5/2 (368.3 eV) and Au4f7/2 (84.0 eV).

An XRD analysis was performed using a Difray-401 X-ray diffractometer (Scientific Instruments Joint Stock Company, Saint Petersburg, Russia) with Bragg–Bretano focusing on Cr*K*_α_ radiation, *λ* = 0.229 nm.

SEM images were taken using a Hitachi TM 3030 scanning electron microscope (Hitachi High-Technologies Corporation, Fukuoka, Japan) with magnification up to 30,000 and a 30 nm resolution and a Zeiss Supra 25 FE-SEM field emission scanning electron microscope (Carl Zeiss AG, Jena, Germany).

The frequency dependence on the conductivity (σ_ac_) was studied using an E7-20 precision LCR-meter (MC Meratest, Moscow, Russia) in the frequency range of 25.0 Hz–1.0 MHz.

DSC thermograms were recorded using a Mettler Toledo DSC823^e^ calorimeter (Giessen, Germany) in the range of 30–350 °C at the rate of 10 °C/min in a nitrogen atmosphere.

TGA thermograms were taken using a Mettler Toledo TGA/DSC1 thermal analyzer (Columbus, OH, USA) in the range of 30–1000 °C in the air and in the argon flow.

## 3. Results and Discussion

### 3.1. Synthesis and Characterization of IR-PAN-a/PDPAC Composites

**Figure 1:**
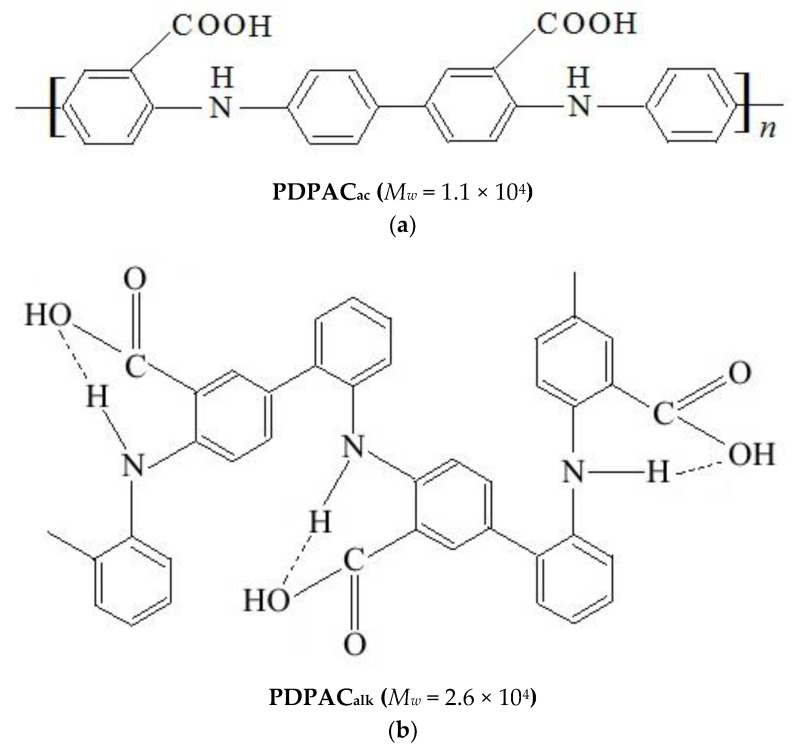
The chemical structure of PDPAC polymers prepared in 5 M H_2_SO_4_ (**a**) and in an NH_4_OH solution in the presence of chloroform (**b**).

The polymer–carbon composites based on PDPAC and IR-PAN-a were prepared using two methods. Hybrid IR-PAN-a/PDPAC materials were synthesized using in situ oxidative polymerization of DPAC in the presence of IR-PAN-a in 5 M H_2_SO_4_ and in an NH_4_OH solution with chloroform. For comparison, polymers of DPAC were obtained under the same conditions. Figure 1 shows the chemical structure of PDPAC depending on the pH of the reaction medium.

**Figure 2:**
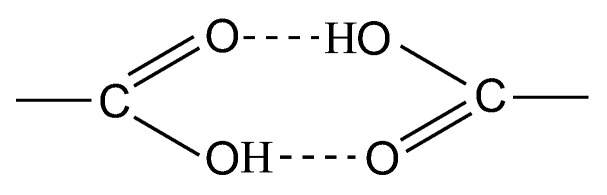
Intermolecular hydrogen bonds between the carboxyl groups in the DPAC monomer.

In the solid phase, dimerization occurs in the DPAC monomer via intermolecular hydrogen bonds between the carboxyl groups (Figure 2). A noticeable broadening in the bands and an increased background absorption in the range of 3600–2400 cm^−1^ are associated with the association of O–H groups.

The dimers of carboxyl groups are destroyed during the polymerization of DPAC in an alkaline medium. The COOH groups (ν_C=О_ = 1675 and 1224 cm^−1^) are associated with the N–H groups (ν_N–H_ = 3175 cm^−1^) of the main chain. The carboxyl groups along the entire polymer chain form intramolecular hydrogen bonds with the amine groups. The absorption band at 3264 cm^−1^ characterizes the associated COOH---N–H carboxyl groups with the hydrogen bond. In the structure of the polymer synthesized in an acidic medium (5 M H_2_SO_4_), the association of carboxyl and amine groups is absent.

IR-PAN-a was obtained under the conditions of PAN pyrolysis in the presence of potassium hydroxide under IR radiation. The originality of the approach lies in the fact that oxidation is applied to PAN which is stabilized at 200 °C and has a disordered structure with a large number of structural defects that the oxygen-containing groups are immobilized on [41,42]. The stabilization stage, firstly, prevents hydrolysis of PAN in an alkaline medium due to the initial structuring in the form of cyclization, and, secondly, oxygen groups placed on the surface of PAN annealed at 200 °C increase the hydrophilicity of the material, which has a beneficial effect on the uniformity of impregnation and distribution of the activating agent in the volume of the material. Subsequent IR pyrolysis at 800 °C leads to the formation of a highly porous N-doped carbon material with a highly developed surface. The carbon structure of IR-PAN-a is characterized by the presence of sp^2^-hybridized carbon atoms due to the formation of graphene planes during heat treatment, as well as of sp^3^-hybridized carbon atoms due to numerous defects in the graphite-like structure in the form of C–O–C, C=O bonds formed during activation in the presence of alkali. The highly porous IR-PAN-a has a spongy structure with cavities ranging in size from 5 to 20 µm, and their walls contain micropores. The specific surface area of the prepared IR-PAN-a reaches 2438 m^2^∙g^−1^ [38].

The formation of the IR-PAN-a/PDPAC composite materials was confirmed using XRD, FTIR, Raman, high-resolution XPS, solid-state CP/MAS (cross-polarization magic angle spinning) ^13^С NMR spectroscopy, and field emission scanning electron microscopy (FE-SEM).

**Figure 3:**
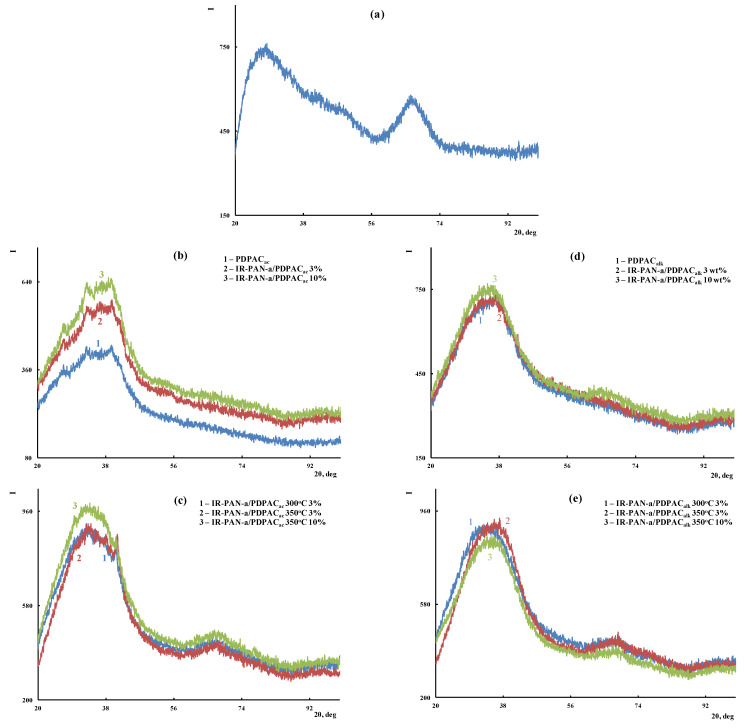
XRD of IR-PAN-a (**a**), PDPAC and IR-PAN-a/PDPAC (**b**,**d**), and IR-PAN-a/PDPAC_IR_ (**c**,**e**), prepared in an acidic (**b**,**c**) and an alkaline medium (**d**,**e**).

According to the XRD analysis, the IR-PAN-a/PDPAC composites are amorphous irrespective of the preparing method (Figure 3).

**Figure 4:**
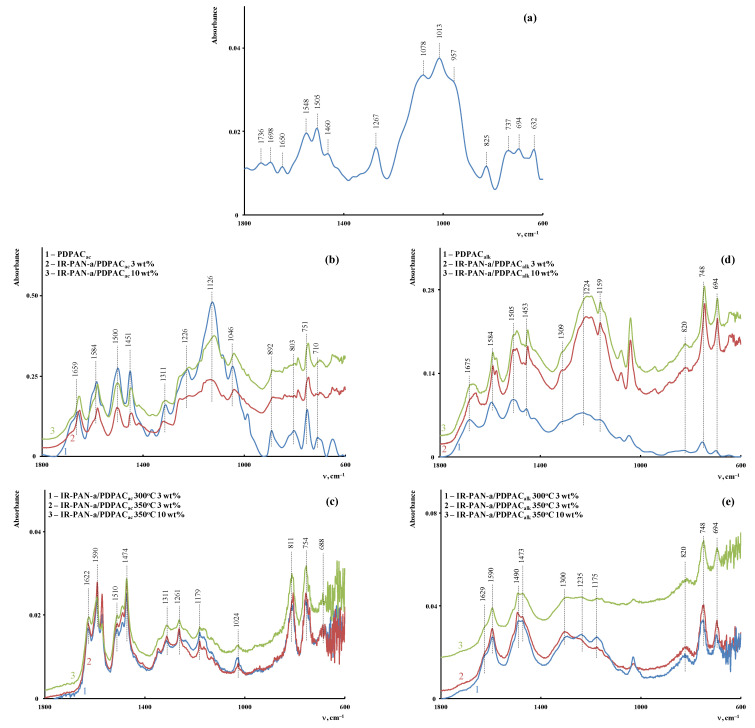
Attenuated total reflection (ATR) FTIR spectra of IR-PAN-a (**a**), PDPAC and IR-PAN-a/PDPAC (**b**,**d**), and IR-PAN-a/PDPAC_IR_ (**c**,**e**), prepared in an acidic (**b**,**c**) and an alkaline medium (**d**,**e**).

Figure 4 shows the ATR FTIR spectra of the composites obtained in an acidic medium (IR-PAN-a/PDPAC_ac_) and an alkaline medium (IR-PAN-a/PDPAC_alk_) before and after IR heating. All the main bands characterizing the chemical structure of PDPAC remain in the FTIR spectra of IR-PAN-a/PDPAC.

As can be seen, the chemical structure of the polymer component has a strong dependency on the pH of the reaction medium for the composite synthesis. During the polymerization of DPAC in an acidic medium (pH 0.3) in the presence of IR-PAN-a, the polymer chain grows via the С–С bonding into the para position of the phenyl rings relative to nitrogen. In the IR-PAN-a/PDPAC_ac_ composite, the absorption bands at 751, 785, and 892 cm^−1^ are due to the out-of-plane bending vibrations of the δ_С–Н_ bonds of the 1,2-, 1,2,4-, and 1,4-substituted benzene rings, respectively. During the polymerization of DPAC in the heterophase system in an alkaline medium (pH 11.4) in the presence of IR-PAN-a, the polymer chain grows via the С–С bonding into the 2- and 4-positions of the phenyl rings relative to nitrogen. In the IR-PAN-a/PDPAC_alk_ composite, the absorption bands at 745 and 820 cm^−1^ correspond to out-of-plane bending vibrations of the δ_С–Н_ bonds of the 1,2-disubstituted and 1,2,4-trisubstituted benzene rings. The shift in the absorption bands, corresponding to stretching vibrations of ν_С–С_ bonds in the aromatic rings indicate the π–π* interaction of PDPAC phenyl rings with the aromatic structures of IR-PAN-a (stacking effect). The charge transfer from the polymer chain to IR-PAN-a is manifested in the shift of skeletal oscillation frequencies of the polymer. The absorption bands at 1652 and 1216 cm^−1^ (IR-PAN-a/PDPAC_ac_) and 1656 and 1211 cm^−1^ (IR-PAN-a/PDPAC_alk_) characterize the stretching vibrations of ν_C=О_ in the COOH groups. A sharp fall in the intensity of these bands in the FTIR spectra of IR-PAN-a/PDPAC_IR_ indicates the removal of carboxyl groups during IR heating of the composites.

**Figure 5:**
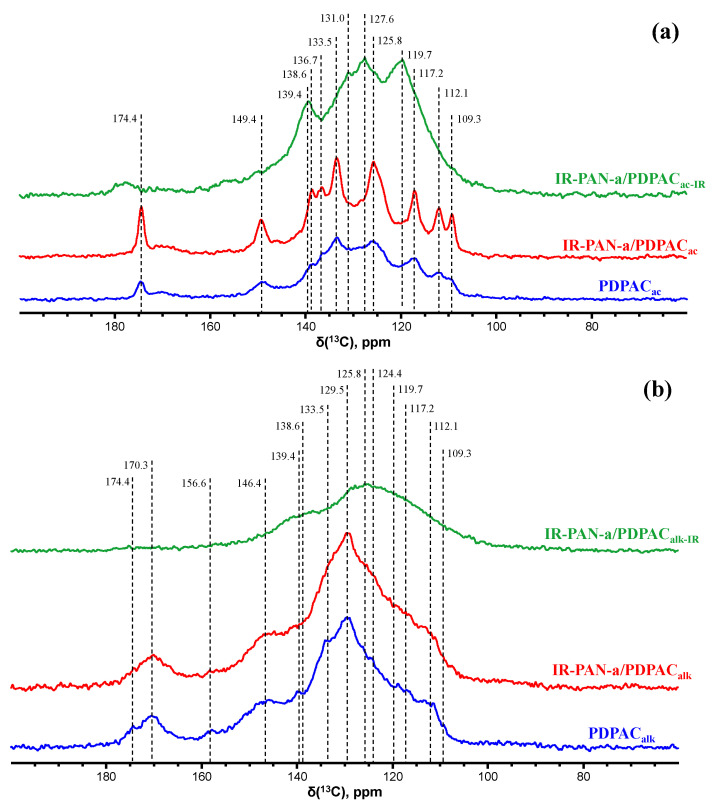
CP/MAS ^13^C NMR spectra of PDPAC, IR-PAN-a/PDPAC, and IR-PAN-a/PDPAC_IR_ prepared in an acidic (**a**) and an alkaline medium (**b**).

Figure 5 shows the CP/MAS ^13^C NMR spectra of the IR-PAN-a/PDPAC composites.

The CP/MAS ^13^C NMR spectrum of the IR-PAN-a/PDPAC_ac_ composite shows an increase in signal intensity at 125.8 and 133.5 ppm compared to the spectrum of the PDPAC_ac_ polymer. The paramagnetic centers of IR-PAN-a reduce the relaxation times T_1_ of carbon atoms in the polymer component due to their interaction. The signal at δ_C_ = 149.4 ppm characterizes the carbon atoms of the C–NH groups. The signal at δ_C_ = 174.4 ppm corresponds to the carboxyl groups.

The solid-state CP/MAS ^13^C NMR spectrum of the IR-PAN-a/PDPAC_alk_ composite retains all signals characterizing the PDPAC_alk_ polymer. Broad signals from 105 to 155 ppm with the maximum at δ_C_ = 129.5 ppm characterize the carbon centers in the benzene rings. The signals in the region of δ_C_ = 146.4 ppm and δ_C_ = 170.3 ppm correspond to carbon atoms of the C–NH and COOH groups, respectively. The broadening of all signals indicates the interaction of the carbon centers with IR-PAN-a, which leads to a decrease in the relaxation time T_1_ of these centers. The overall broadening of the signals also confirms indirectly the low degree of crystallinity in the composite, which is consistent with the XRD data (Figure 3).

In the solid-state CP/MAS ^13^C NMR spectra of IR-PAN-a/PDPAC_IR_ composites, a sharp decrease in signal intensity at δ_C_ = 149.4 ppm and δ_C_ = 174.4 ppm (IR-PAN-a/PDPAC_ac_) and δ_C_ = 146.4 ppm and δ_C_ = 170.3 ppm (IR-PAN-a/PDPAC_alk_) characterizing the C–NH and COOH groups is explained by the fact that IR heating of composites at 300–350 °C causes dehydrogenation of phenylenamine structures with the formation of C=N bonds, which leads to partial removal of the carboxyl groups.

**Figure 6:**
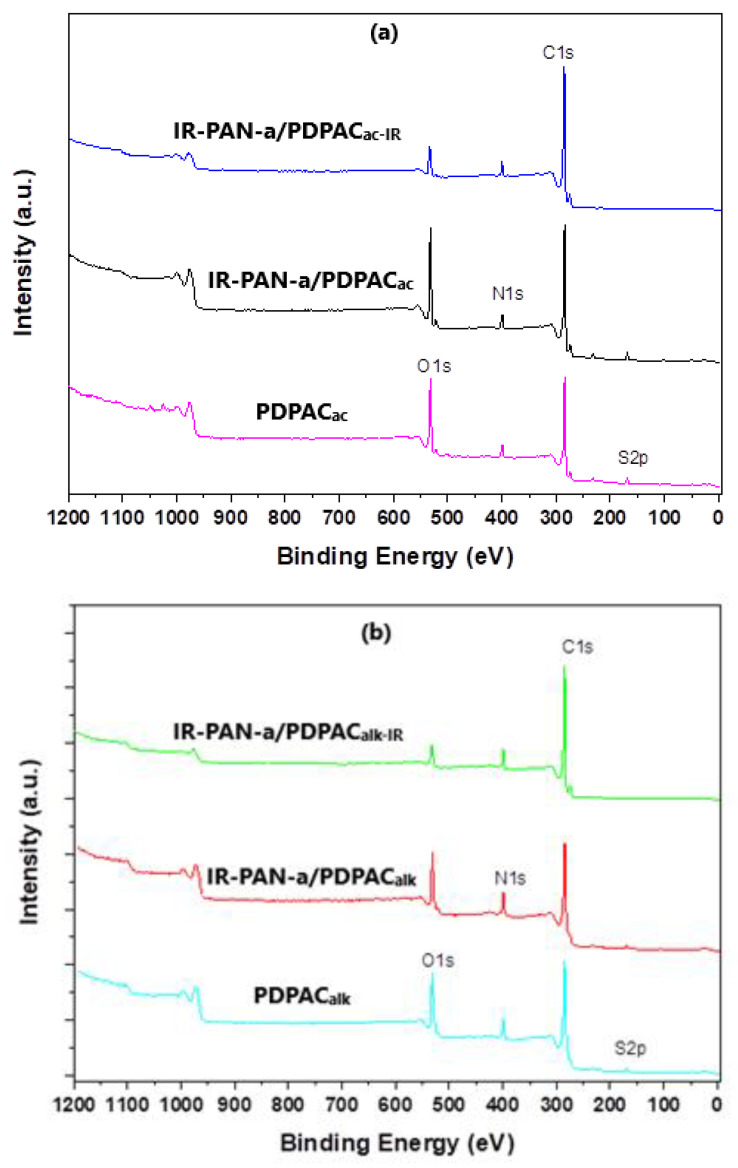
XPS survey spectra of PDPAC, IR-PAN-a/PDPAC, and IR-PAN-a/PDPAC_IR_ prepared in an acidic (**a**) and an alkaline medium (**b**).

**Figure 7:**
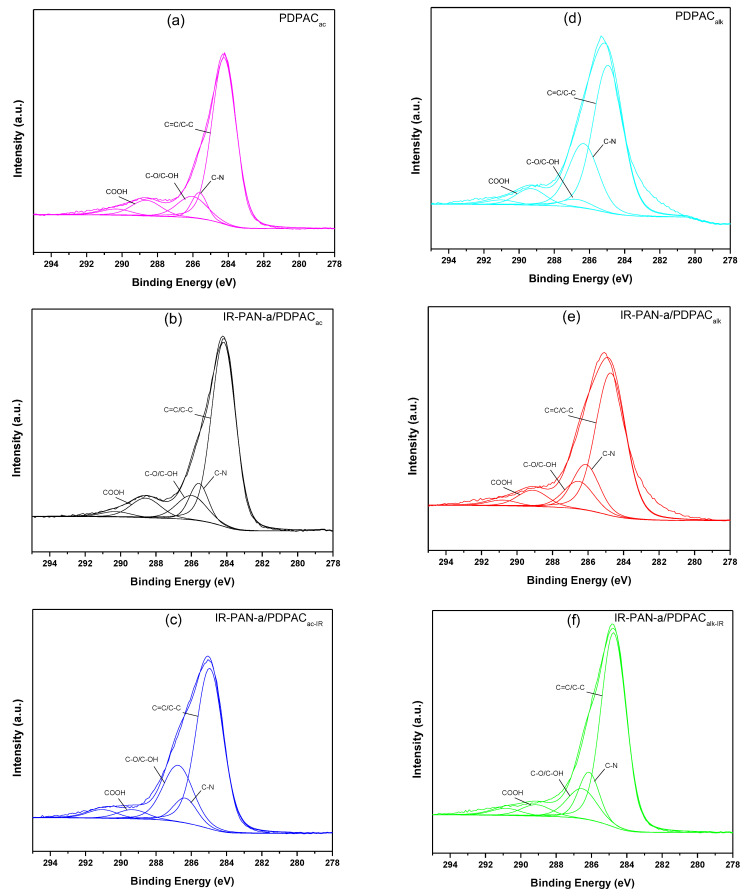
C1s XPS spectra of PDPAC (**a**,**d**), IR-PAN-a/PDPAC (**b**,**e**), and IR-PAN-a/PDPAC_IR_ (**c**,**f**) prepared in an acidic (**a**–**c**) and an alkaline medium (**d**–**f**).

**Figure 8:**
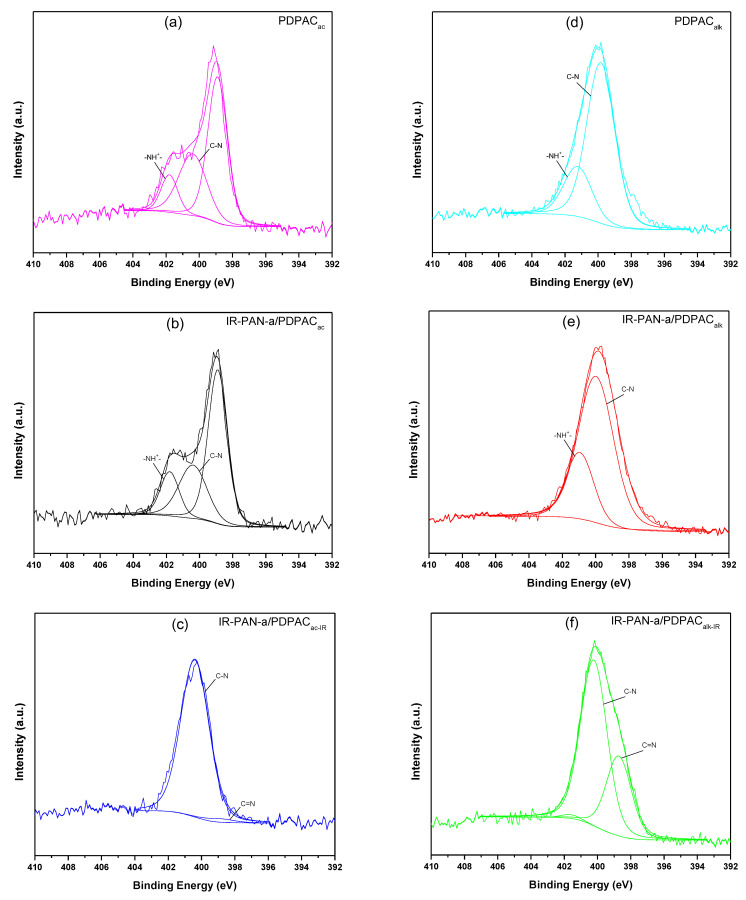
N1s XPS spectra of PDPAC (**a**,**d**), IR-PAN-a/PDPAC (**b**,**e**), and IR-PAN-a/PDPAC_IR_ (**c**,**f**) prepared in an acidic (**a**–**c**) and an alkaline medium (**d**–**f**).

Decarboxylation of the polymer chain and the formation of C=N bonds are confirmed using the XPS data. The XPS spectra were recorded to analyze the electronic structure and chemical bond information of surface elements. As shown in Figure 6, the survey XPS spectra of PDPAC and IR-PAN-a/PDPAC show peaks for C 1s, O 1s, N 1s, S 1s, and S 2p. The C, O, N, and S content and binding energy are given in Table 1. The core-level spectrum of C 1s can be deconvoluted into four peaks at 284 eV (C=C/C-C), 285 eV (C-N), 286 eV (C-O/C-OH), and 289 eV (C-OOH) (Figure 7). A weak peak at 290 eV corresponds to the π–π* transition. After the resulting composites are treated with IR radiation, the intensity of the peak at 289 eV, typical of the COOH group [34], decreases in the C1s XPS spectra of IR-PAN-a/PDPAC_IR_. The share of COOH groups drops from 8.86% to 3.86% (in acid) and from 7.00% to 4.67% (in alkali). At the same time, the oxygen content in the IR-heated composites decreases from 18.26% to 8.05% (in acid) and from 15.33% to 7.16% (in alkali). In the N1s XPS spectra of IR-PAN-a/PDPAC_IR_ (Figure 8), a peak appears at 398.72 eV, corresponding to the C=N binding energy [43,44,45]. The peak at 401 eV characterizing the -NH^+^- binding energy [34] is absent in the N1s XPS spectra of IR-PAN-a/PDPAC_IR_. The absence of S 1s and S 2p peaks in the survey XPS spectra of IR-PAN-a/PDPAC_IR_ is associated with the removal of the dopant (HSO_4_^–^) during IR heating. As can be seen in Figure 8, the N1s XPS spectra of PDPAC_ac_ and IR-PAN-a/PDPAC_ac_ in conductive form can be deconvoluted into three peaks at 398.9, 400.4, and 401.8 eV.

**Figure 9:**
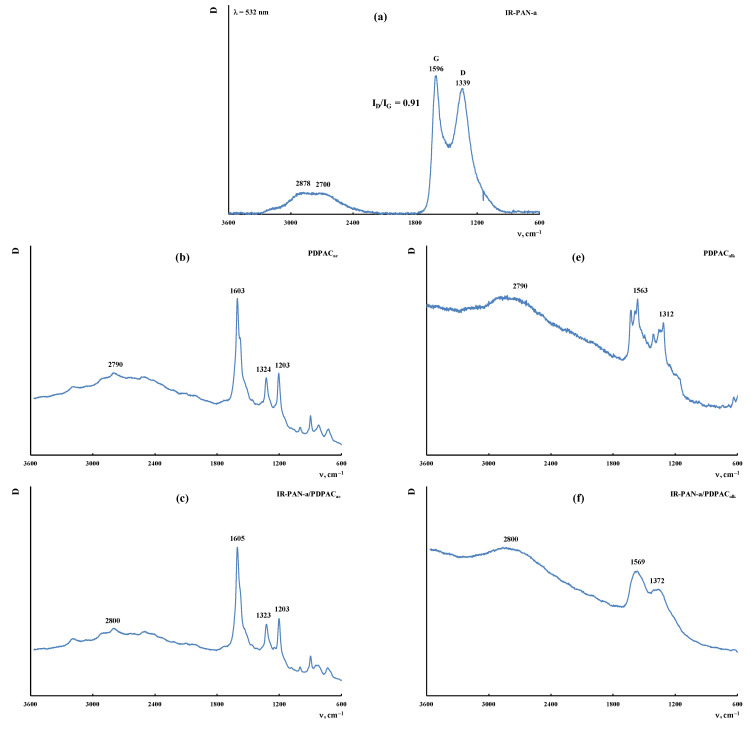

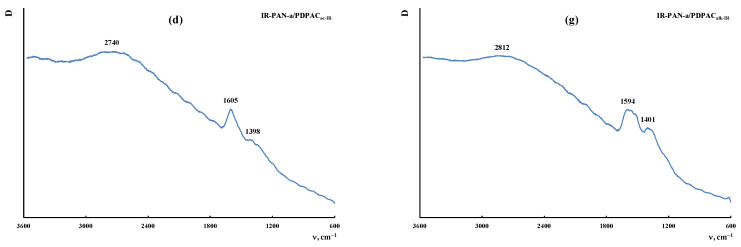
Raman spectra of IR-PAN-a (**a**), PDPAC (**b**,**e**), IR-PAN-a/PDPAC (**c**,**f**), and IR-PAN-a/PDPAC_IR_ (**d**,**g**) prepared in an acidic (**b**–**d**) and an alkaline medium (**e**–**g**).

Figure 9 shows the Raman spectra of the IR-PAN-a, PDPAC, and IR-PAN-a/PDPAC composites prepared using two methods before and after IR heating. As can be seen, in the Raman spectrum of IR-PAN-a, there are two pronounced bands: a G band at ~1596 cm^−1^ from sp^2^ carbon atoms and a D band at ~1339 cm^−1^ from sp^3^ carbon atoms. The G band is a distinctive feature of graphite structures, whereas the D band is associated with disordered and defective structures [43,45]. The intensity ratio of these bands in the IR-PAN-a Raman spectrum is I_D_/I_G_ = 0.91. The splitting of the G and D bands in the Raman spectra of the IR-PAN-a/PDPAC composites is associated with the presence of a polymer component. The intensity ratio of the I_D_/I_G_ in the IR-heated composites decreases from 0.76 to 0.73 (IR-PAN-a/PDPAC_ac-IR_) and from 0.87 to 0.82 (IR-PAN-a/PDPAC_alk-IR_), which is associated with the dehydrogenation of phenylenamine structures of the polymer component with the formation of sp^2^ carbon atoms.

**Figure 10:**
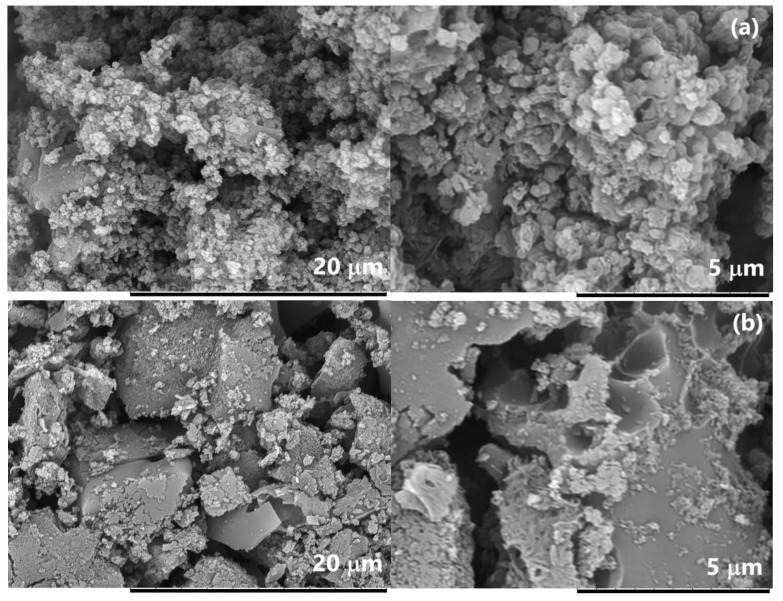
SEM images of IR-PAN-a/PDPAC_ac_ (**a**) and IR-PAN-a/PDPAC_alk_ (**b**).

**Figure 11:**
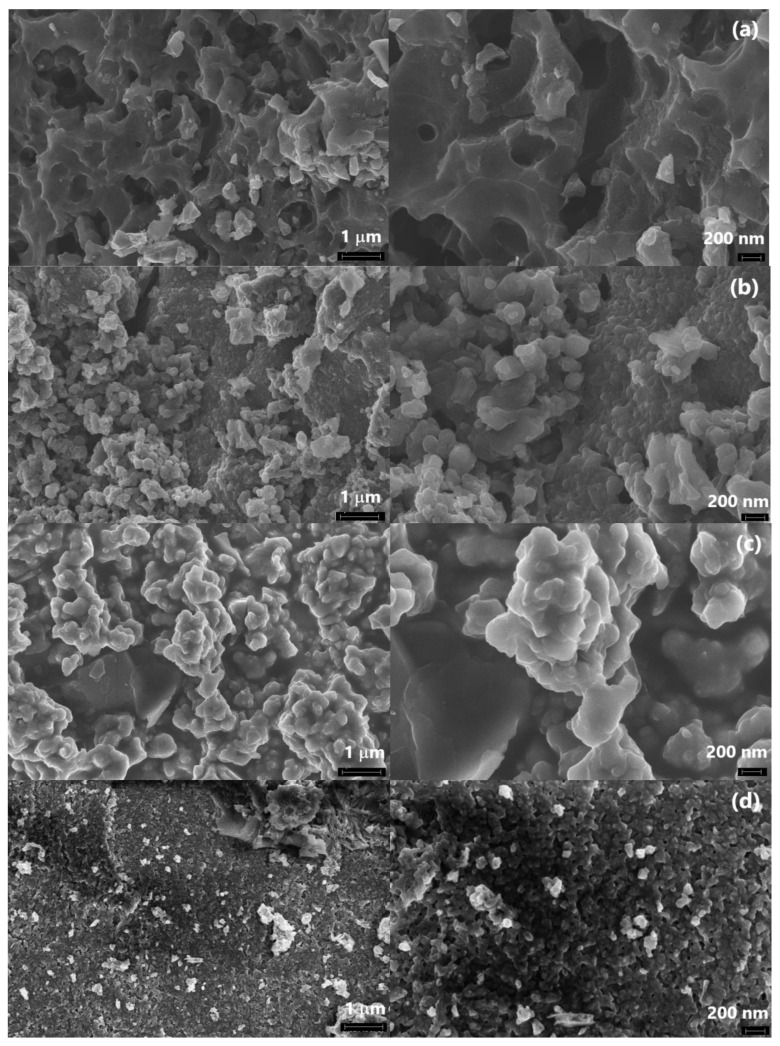

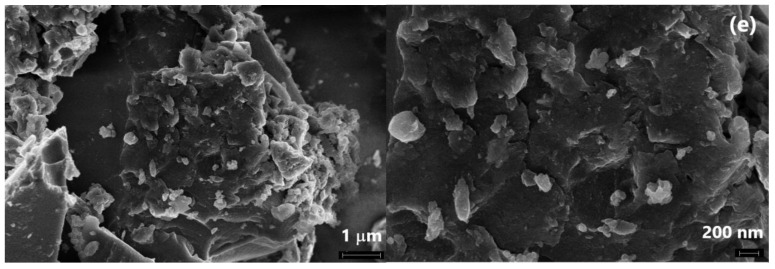
FE-SEM images of IR-PAN-a (**a**), IR-PAN-a/PDPAC_ac_ (**b**), IR-PAN-a/PDPAC_alk_ (**c**), IR-PAN-a/PDPAC_ac-IR_ (**d**), and IR-PAN-a/PDPAC_alk-IR_ (**e**).

Figure 10 and Figure 11 show electron microscopic images of the IR-PAN-a/PDPAC composites. As can be seen, the morphology of the composites depends on the pH of the synthesis reaction medium. IR-PAN-a/PDPAC_ac_ has a globular structure, whereas in IR-PAN-a/PDPAC_alk_, cavities are formed in places of chloroform drops (Figure 10).

According to the FE-SEM data, during the synthesis of the composites, the spongy structure of IR-PAN-a is filled with a monomer solution followed by the formation of a polymer layer on the surface of the carbon material (Figure 11).

### 3.2. Thermal Properties of Materials

**Figure 12:**
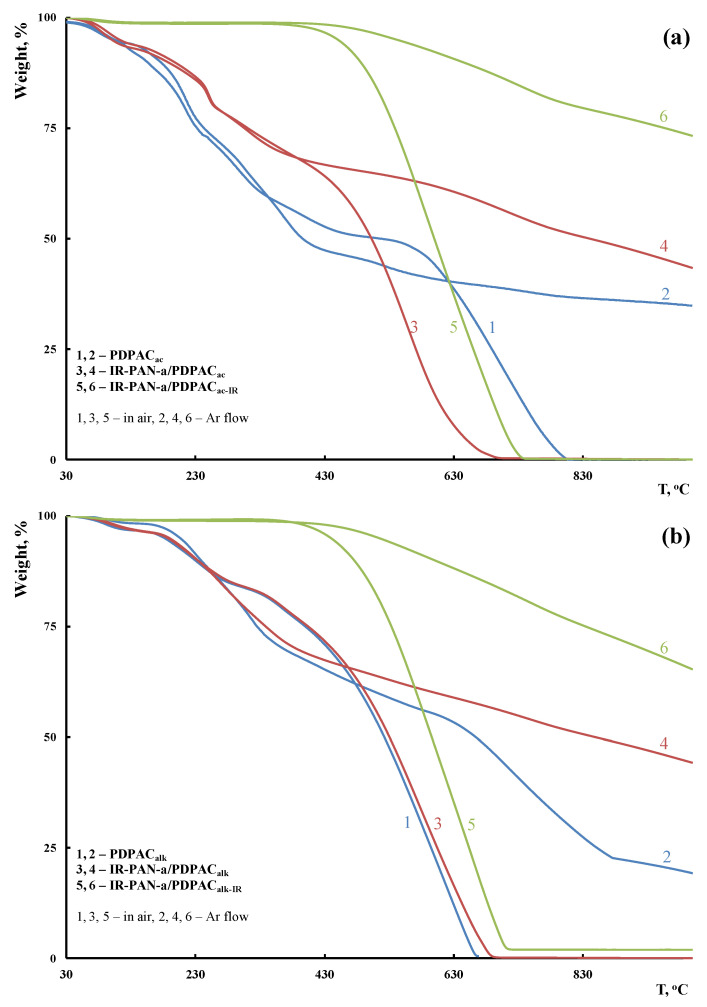
TGA thermograms of PDPAC (1, 2), IR-PAN-a/PDPAC (3, 4), and IR-PAN-a/PDPAC_IR_ (5, 6) prepared in an acidic (**a**) and an alkaline medium (**b**) at heating of up to 1000 °C in the Ar flow (1, 3, 5) and in the air (2, 4, 6).

TGA, DSC, and DTG methods were used to study the thermal stability of the hybrid IR-PAN-a/PDPAC composites depending on the synthesis method. The composite materials were IR-heated to reduce the oxygen content and enhance their thermal stability. The IR heating temperature was determined using the TGA data to prevent polymer chain degradation. Figure 12 shows TGA thermograms of IR-PAN-a/PDPAC compared to PDPAC when heated up to 1000 °C in the argon flow and in the air. The content of activated carbon in the composites is C_IR-PAN-a_ = 3 wt % relative to the monomer weight. Table 2 lists the main thermal characteristics of the materials.

**Figure 13:**
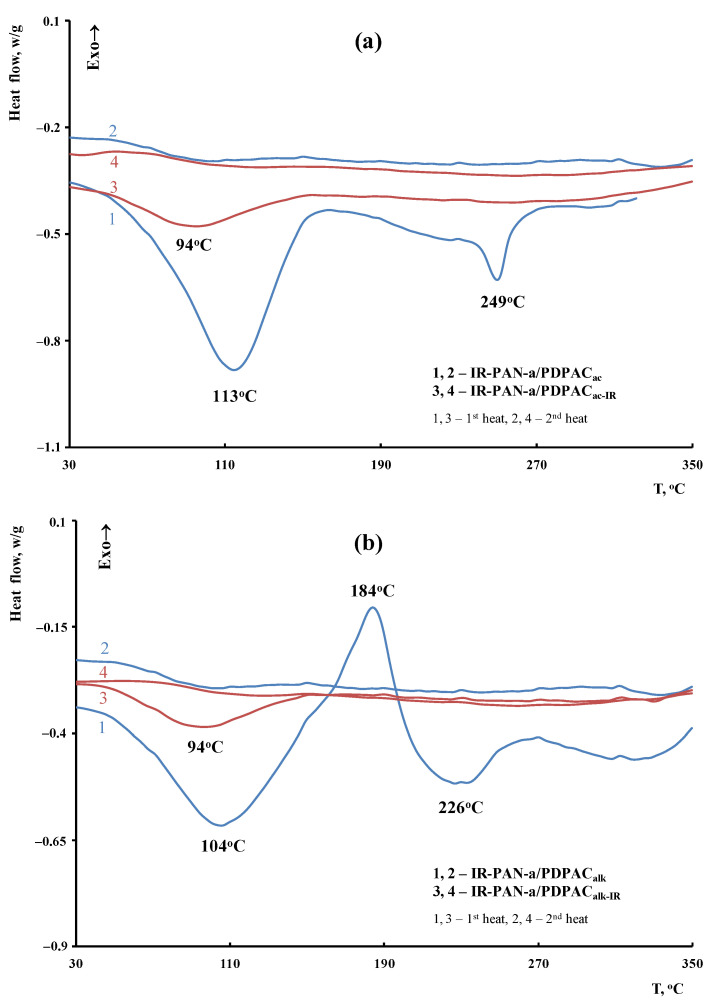
DSC thermograms of IR-PAN-a/PDPAC (1, 2) and IR-PAN-a/PDPAC_IR_ (3, 4) prepared in an acidic (**a**) and an alkaline medium (**b**), upon heating in the nitrogen flow to 350 °C (1, 3—first heating, 2, 4—second heating).

The weight loss at low temperatures is associated with the removal of moisture. The DSC thermograms of the IR-PAN-a/PDPAC composites demonstrate an endothermic peak at ~104–113 °C (Figure 13). An endothermic peak at 226–249 °C is associated with the partial removal of the COOH groups that takes place when the IR-PAN-a/PDPAC composites are heated.

As seen in Figure 12, IR heating of the obtained materials leads to a significant increase in their thermal properties. The DSC thermograms of IR-PAN-a/PDPAC_IR_ do not show thermal effects up to 350 °С. The degradation processes of the IR-heated composites begin at temperatures above 430 °С.

**Figure 14:**
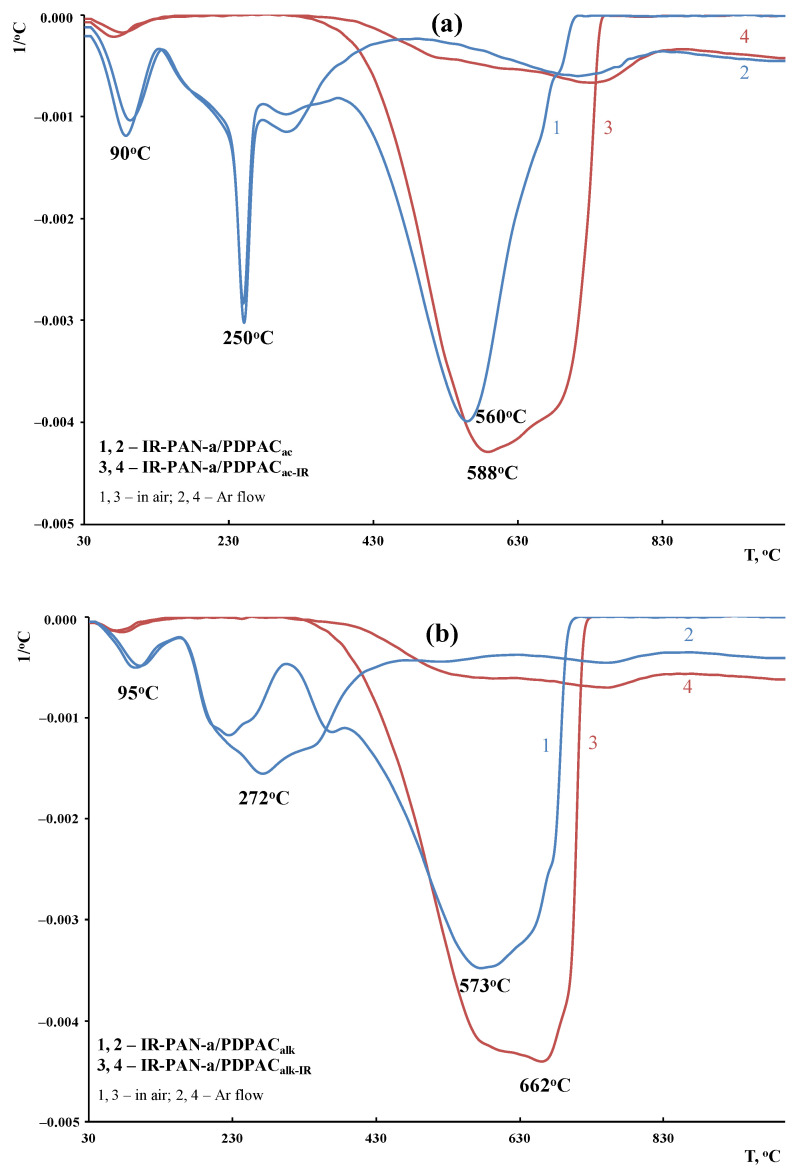
DTG curves of IR-PAN-a/PDPAC (1, 2) and IR-PAN-a/PDPAC_IR_ (3, 4) prepared in an acidic (**a**) and an alkaline medium (**b**), in the Ar flow (1, 3) and in the air (2, 4).

According to DTG, the decomposition processes of the composites before and after IR heating occur within the range of 340–750 °C, with the maxima at 560 and 588 °C (in an acidic medium) and 573 and 662 °C (in an alkaline medium) (Figure 14). The IR-heated composites lose half of the original weight in an inert atmosphere at temperatures above 1000 °C, whereas for IR-PAN-a/PDPAC, the temperature range is 840–849 °C. At 1000 °C the residue is 65–73% in the IR-PAN-a/PDPAC_IR_ composites.

### 3.3. Electrical Characterization of Materials

**Figure 15:**
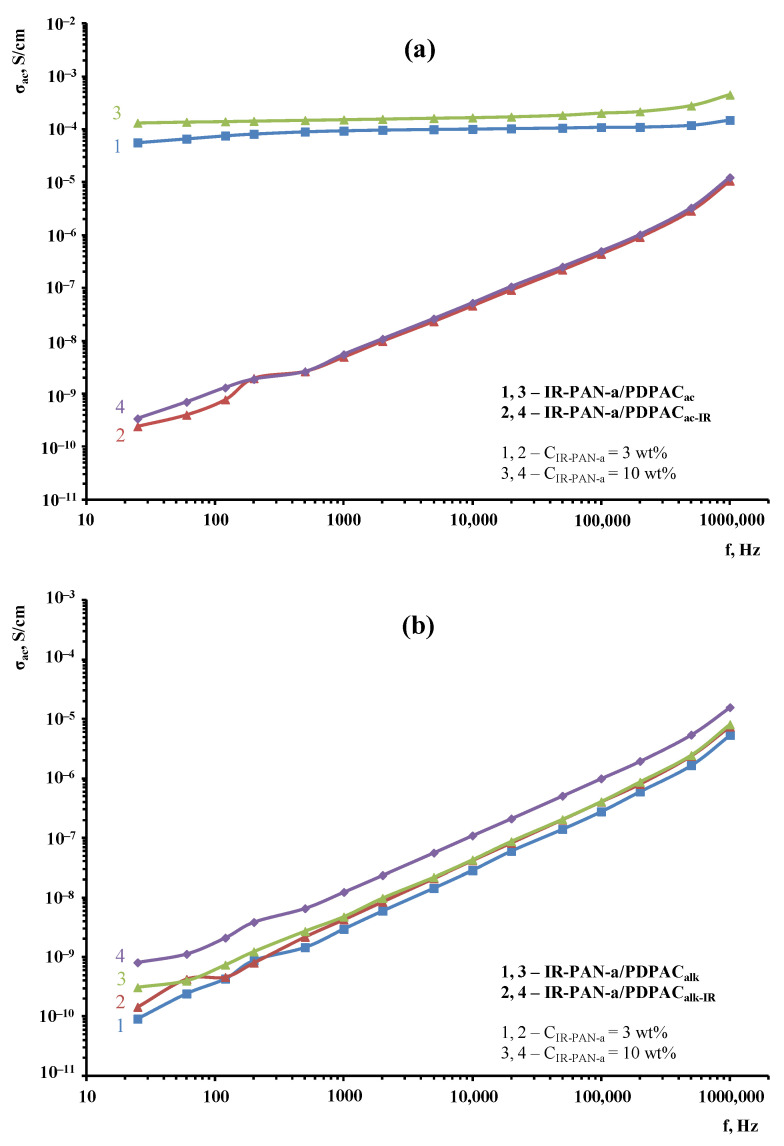
Frequency dependence on the conductivity for IR-PAN-a/PDPAC (1, 3) and IR-PAN-a/PDPAC_IR_ (2, 4) prepared at C_IR-PAN-a_ = 3 wt % (1, 2) and 10 wt % (3, 4) in an acidic (**a**) and an alkaline medium (**b**).

Figure 15 demonstrates the frequency dependences on the *ac* conductivity (σ_ac_) for the IR-PAN-a/PDPAC composites prepared using two methods before and after IR heating. Table 3 shows the main conductivity values of materials calculated using the equation for the dependence of conductivity on the frequency:

σ*_ac_* = σ*_dc_* + *Aω^n^*

As seen, regardless of the method of synthesis, the electrical conductivity of the IR-PAN-a-based composites has little dependence on the carbon material concentration at C_IR-PAN-a_ = 3 and 10 wt %. The value of *n* = 0.30–0.999 calculated using the equation of the frequency dependence on the electrical conductivity indicates the hopping conductivity mechanism (0 ≤ *n* ≤ 1) [46,47].

The IR-PAN-a/PDPAC_ac_ composites show weak dependence of the conductivity σ_ac_ on frequency. In the frequency range of 25–10^6^ Hz, the *ac* conductivity of the composites increases only from 1.3 × 10^–5^ to 4.5 × 10^–5^ S/cm. The weak frequency dependence on the *ac* conductivity is due to the fact that the composites have passed their percolation threshold [48]. During the composite synthesis in an acidic medium, doping of the polymer component occurs, which makes the main contribution to the composite conductivity. A sharp drop in the electrical conductivity of the IR-PAN-a/PDPAC_ac-IR_ composites to 3.4 × 10^–10^ S/cm is associated with the removal of the dopant (HSO_4_^−^) during IR heating.

As can be seen in Figure 15, the IR-PAN-a/PDPAC_alk_ composites prepared before and after IR heating show a gradual increase in electrical conductivity. In the frequency range of 25–10^6^ Hz, the electrical conductivity of the materials increases by five orders of magnitude to 1.6 × 10^–5^ S/cm.

## 4. Conclusions

Polymer–carbon composites based on PDPAC and IR-PAN-a were synthesized for the first time using in situ oxidative polymerization in an acidic medium and in the heterophase system in an alkaline medium. IR-PAN-a with a hierarchical pore structure is characterized by a highly developed surface. IR-PAN-a was prepared using pyrolysis of PAN in the presence of KOH under IR radiation. The dependence of the chemical structure and morphology of the polymer matrix on the pH of the reaction medium of composite synthesis was shown. The resulting composites were IR-heated to reduce the oxygen content and enhance their physical and chemical properties. The IR-PAN-a/PDPAC composites are thermally stable and electrically conductive. The electrical properties stem from the nature of the polymer component. In the low-frequency range, due to polymer chain doping, the conductivity of IR-PAN-a/PDPAC_ac_ is significantly higher (by five orders of magnitude) than the conductivity of IR-PAN-a/PDPAC_alk_. At 1000 °C in an inert atmosphere, the residue is 65–73% in the IR-PAN-a/PDPAC_IR_ composites. The prepared hybrid materials can find applications in the field of electrochemical current power supplies, low temperature fuel cells, supercapacitors, etc.

## Figures and Tables

**Table 1:** XPS study of materials.

Element	PDPAC_ac_			PDPAC_alk_			Bonds
	C_at_, at %	BE, eV	Line Intensity %	C_at_, at %	BE, eV	Line Intensity %	
С1	71.82	284.24	70.35	74.90	284.94	61.15	С=С/C-C
С2		285.64	7.01		286.34	26.75	С-N
С3		286.04	11.18		286.74	3.26	С-О/C-OH
C4		288.64	8.20		289.34	6.87	COOH
С5		290.34	3.26		291.04	1.97	π–π* transition
N1	6.27	398.90	54.73	8.11	-	-	С=N
N2		400.40	33.27		399.83	76.28	С-N
N3		401.80	11.99		401.23	23.72	-NH^+^-
O1	19.19	531.57	54.5	15.46	531.43	55.91	C-O
O2		533.11	36.73		533.10	44.09	O-H
O3		535.83	8.77		-	-	O-H (H_2_O)
S	2.71			1.53			
	IR-PAN-a/PDPAC_ac_			IR-PAN-a/PDPAC_alk_			
С1	74.49	284.18	67.09	74.05	284.72	61.27	С=С/C-C
С2		285.58	10.66		286.12	17.35	С-N
С3		285.98	10.99		286.52	11.89	С-О/C-OH
C4		288.58	8.86		289.12	7.00	COOH
С5		290.28	2.40		290.82	2.49	π–π* transition
N1	4.90	398.90	56.13	9.30	-	-	С=N
N2		400.40	28.36		400.19	70.80	С-N
N3		401.80	15.51		401.23	29.20	-NH^+^-
O1	18.26	531.45	52.50	15.33	530.98	51.89	C-O
O2		532.96	47.50		532.70	41.38	O-H
O3		-	-		534.80	6.72	O-H (H_2_O)
S	2.34			1.32			
	IR-PAN-a/PDPAC_ac-IR_ *			IR-PAN-a/PDPAC_ac-IR_ *			
С1	87.29	284.95	60.60	84.81	284.74	66.66	С=С/C-C
С2		286.35	8.20		286.14	13.49	С-N
С3		286.75	23.69		286.54	12.26	С-О/C-OH
C4		289.35	3.86		289.14	4.67	COOH
С5		291.05	3.64		290.84	2.91	π–π* transition
N1	4.66	398.90	2.19	8.03	398.72	31.09	С=N
N2		400.40	97.81		400.22	68.91	С-N
N3		-	-		-	-	-NH^+^-
O1	8.05	531.46	25.42	7.16	531.49	36.92	C-O
O2		533.54	38.50		533.49	45.92	O-H
O3		535.26	36.07		536.00	17.15	O-H (H_2_O)
S	-			-			

* IR heating at 350 °C.

**Table 2:** Thermal properties of materials.

Materials	Property			
	* *T*_5%_, °C	** *T*_50%_, °C	Weight Loss at 350 °C, %	*** Residue, %
PDPAC_ac_	104/102	517/396	41/42	35
IR-PAN-a/PDPAC_ac_	101/113	503/840	29/30	43
IR-PAN-a/PDPAC_ac-IR_^^^	451/543	600/>1000	2/1	73
PDPAC_alk_	185/205	523/663	20/29	20
IR-PAN-a/PDPAC_alk_	188/190	530/849	19/26	44
IR-PAN-a/PDPAC_alk-IR_^^^	440/515	596/>1000	1/1	65

* *T*_5%_, ** *T*_50%_—5 and 50% weight losses (Air/Ar), *** residue at 1000 °C (Ar). ^ IR heating at 350 °C.

**Table 3:** The conductivity values of materials.

Materials	[IR-PAN-a], wt %	σ_ac_, S/cm		* σ_dc_, S/cm	** n	*** A
		at 25 Hz	at 10^6^ Hz			
PDPAC_ac_	–	1.4 × 10^–5^	2.1 × 10^–5^	1.0 × 10^–5^	0.45	6.9 × 10^–9^
IR-PAN-a/PDPAC_ac_	3	5.6 × 10^–5^	1.5 × 10^–4^	9.1 × 10^–5^	0.30	3.2 × 10^–7^
	10	1.3 × 10^–5^	4.5 × 10^–5^	1.1 × 10^–5^	0.53	4.7 × 10^–9^
IR-PAN-a/PDPAC_ac-IR_^^^	3	2.5 × 10^–10^	1.1 × 10^–5^	1.9 × 10^–11^	0.74	4.6 × 10^–11^
	10	3.4 × 10^–10^	1.3 × 10^–5^	3.6 × 10^–11^	0.99	1.4 × 10^–12^
PDPAC_alk_	–	8.8 × 10^–11^	1.2 × 10^–7^	2.8 × 10^–12^	0.75	8.5 × 10^–12^
IR-PAN-a/PDPAC_alk_	3	9.1 × 10^–11^	5.4 × 10^–6^	0.7 × 10^–11^	0.996	5.7 × 10^–13^
	10	1.5 × 10^–10^	7.4 × 10^–6^	0.8 × 10^–11^	0.99	6.5 × 10^–13^
IR-PAN-a/PDPAC_alk-IR_ ^	3	3.1 × 10^–10^	8.1 × 10^–6^	1.8 × 10^–11^	0.73	4.8 × 10^–11^
	10	8.1 × 10^–10^	1.6 × 10^–5^	6.8 × 10^–11^	0.999	2.3 × 10^–12^

^ IR heating at 350 °C; * σ_dc_—the frequency independent (dc) part of conductivity; ** n—the exponential parameter (0 ≤ *n* ≤ 1); *** A—the thermally activated quantity.

## Data Availability

Not applicable.
